# Minnesota State Dental Society

**Published:** 1887-08

**Authors:** T. E. Weeks


### MINNESOTA STATE DENTAL SOCIETY.

BY T. E. WEEKS, D. D. S.

The annual meeting of the Minnesota State Dental Society, held in Minneapolis, commencing July 13th, was a successful one from every point of view. About seventy-five members were in attendance, and the papers and discussions were especially interesting and profitable.

Owing to the absence of the chairman of the committee, nothing was done regarding the Black testimonial matter, save to continue the committee and to instruct them to do all that it is possible to do by correspondence.

Dr. H. L. Crittenden, of Northfield, presented the society with a handsome and unique gavel of hard rubber, fashioned like a superior molar tooth, mounted in gold, with handle of ebony, suitably inscribed. It was voted that it be loaned to the Dental Section of the Medical Congress for the use of the chairman at its coming meeting.

Dr. L. P. Haskell, of Chicago, was present and gave a clinic in continuous gum work.

Dr. A. C. Hewett, of Chicago, illustrated the Herbst method of filling teeth, using the Hewett matrix.

Dr. E. B. Call, of Peoria, Ill., illustrated his method of filling teeth by lining the walls with soft gold, tin, or tin and gold, folding some of the material over one of the Call matrices before its insertion, and then condensing the filling into the cavity and against the margin.

Dr. W. A. Spaulding filled a mesial cavity, using Steurer's gold.

Drs. M. G. Jennison and L. C. Gould operated for necrosis, caused by an impacted wisdom tooth.

Dr. C. H. Goodrich filled a distal cavity in a second bicuspid, without using the matrix.

Dr. L. D. Leonard read a paper upon "Lesions of the Mouth," illustrated by views shown through the stereopticon, of slides representing embryonic and pathological conditions.

Dr. E. H. Angle presented "Notes on Orthodontia, together with a New System of Regulating and Retaining Appliances."

Dr. F. H. Brimmer presented his process of obtunding sensitive dentine by vibration. (See Dr. Brimmer's paper in this journal, page 301.)

Dr. Hewett presented a paper upon the Herbst process, and a number of interesting cases were reported.

A committee was appointed to collect and take charge of a fund, to be raised by requesting each member to contribute five dollars, to be expended in resisting unjust patents.

Some idea of the interest manifested in the meeting may be gained from a knowledge of the fact that seven sessions were held, with two half-day clinics; that the thermometer did not go below 80 degrees at any time during the day or night, and yet not one of the members in attendance upon the meeting was absent from any session, unless it were the opening and closing.

The following were elected officers to serve during the ensuing year:

*President*—Dr. H. L. Crittenden, Northfield.
*Vice-President*—E. H. Angle, D. D. S., Minneapolis.
*Recording Secretary*—Dr. D. W. Edwards, Le Seuer.
*Corresponding Secretary*—Dr. L. C. Gould, St. Paul.
*Treasurer*—H. M. Reid, D. D. S., Minneapolis.

